# Quiet Quitting and Professional Burnout: Contemporary Challenges for Nursing Leadership

**DOI:** 10.3390/nursrep16040140

**Published:** 2026-04-15

**Authors:** João Miguel Almeida Ventura-Silva, Olga Maria Pimenta Lopes Ribeiro, Elaine Cristina Novatzki Forte, Letícia de Lima Trindade, Susana Filipa Mendes Castro, Marlene Patrícia Ribeiro, Diana Moreira Sanches, Sónia Cristina Costa Barros, Irina Alexandra Lopes Almeida, David Rigor Lage, Samuel Spiegelberg Züge

**Affiliations:** 1 Department of Nursing, Northern Health School of the Portuguese Red Cross, 3720-126 Oliveira de Azeméis, Portugal; david.r.lage@azores.gov.pt; 2 RISE-Health, 4200-319 Porto, Portugal; 3 RISE-Health, Nursing School, University of Porto, 4200-072 Porto, Portugal; olgaribeiro@esenf.pt; 4 Department of Nursing, Federal University of Santa Catarina, Florianópolis 88040-900, Brazil; elaine.forte@ufsc.br; 5 Department of Nursing, University of the State of Santa Catarina, Chapecó 89815-630, Brazil; leticia.trindade@udesc.br; 6 Department of Nursing, Portuguese Oncology Institute of Porto Francisco Gentil, 4200-072 Porto, Portugal; susanafcastro@ipoporto.min-saude.pt; 7 Abel Salazar Institute of Biomedical Sciences, University of Porto, 4050-313 Porto, Portugal; marlene.ribeiro@ulsts.min-saude.pt; 8 Department of Nursing, Tamega and Sousa Local Health Unit, 4564-007 Penafiel, Portugal; 9 Department of Nursing, Gaia and Espinho Local Health Unit, 4434-502 Vila Nova de Gaia, Portugal; diana.sanches@ulsge.min-saude.pt; 10 Department of Nursing, São João Local Health Unit, 4200-319 Porto, Portugal; sonia.barros@ulssjoao.min-saude.pt; 11 Department of Community Medicine, Information and Health Decision Sciences (MEDCIDS), Faculty of Medicine, University of Porto, 4099-002 Porto, Portugal; 12 Department of Health Sciences, Jean Piaget Higher Polytechnic Institute of Benguela, Benguela 1393, Angola; irina.almeida@unipiaget-angola.org; 13 Department of Nursing, Santo Espírito Hospital on Terceira Island, 9700-049 Angra do Heroísmo, Portugal; 14 Department of Nursing, Chapecó Region Community University, Chapecó 89809-900, Brazil; samuel.zuge@unochapeco.edu.br

**Keywords:** burnout, intention to leave the profession, occupational stress, leadership

## Abstract

**Objectives**: This study aimed to evaluate the relationship between quiet quitting and burnout among nurses, considering the influence of sociodemographic and occupational factors in healthcare settings. **Methods**: This cross-sectional, observational, and quantitative study was conducted from April to July 2025 in health services in northern Portugal, involving 1097 nurses who completed a questionnaire, the Silent Employment Abandonment Scale, and the Shirom–Melamed Burnout Scale. Descriptive and inferential analyses examined associations between sociodemographic variables, work context, and outcomes. **Results**: The 1097 participating nurses showed a positive correlation between overall quiet quitting and total burnout across all domains. Burnout remained significantly associated with overall quiet quitting after adjustment, and physical and cognitive fatigue showed the most consistent independent associations across models. The strongest coefficients were observed for the ‘lack of motivation’ dimension. **Conclusions**: Quiet quitting was consistently associated with burnout among nurses after adjustment for sociodemographic and occupational factors. The pattern of results was stronger for physical and cognitive fatigue and for lack of motivation, supporting the interpretation of quiet quitting as an important correlation of occupational strain in nursing and reinforcing the need for organizational and leadership strategies that reduce fatigue and sustain professional engagement.

## 1. Introduction

In recent years, quiet quitting has gained visibility in the workplace as a pattern where employees remain in their jobs while reducing engagement and effort beyond what is expected, limiting their performance to formal job demands and minimizing contributions beyond essential duties [1,2]. In other words, it involves limiting work activities to the minimum needed to keep employment [3], representing a subtler form of disengagement than traditional turnover, as employees voluntarily reduce their productivity and performance [4]. Since quiet quitting is defined as a distancing attitude toward professional activities, characterized by disinterest, lack of initiative, and reduced motivation [5], it is important to note that this phenomenon should not be confused with disengagement at work, presenteeism, withdrawal behaviors, or the cynicism associated with burnout. Presenteeism primarily refers to staying at work despite illness, while withdrawal behaviors include manifestations such as tardiness, absenteeism, or behaviors linked to the intention to leave. Cynicism, on the other hand, reflects a negative cognitive-affective attitude toward work. In contrast, quiet quitting involves choosing to limit professional investment to fulfill the job’s formal requirements [6,7,8].

In nursing, this phenomenon is especially important because care delivery relies not only on technical skills but also on ongoing effort, effective coordination, and relational commitment with patients and teams, elements that support continuity of care, team performance, and patient safety [5]. In hospital settings, quiet quitting among nurses has been mainly linked to high workload, staff shortages, low recognition, and insufficient managerial support, leading to adherence to the “minimum necessary” and a decline in initiative and motivation [9]. Furthermore, fatigue, stress, and toxic organizational environments may lead to disengagement, with potential outcomes such as decreased quality of care, team conflicts, and an increased likelihood of turnover intentions.

A study of healthcare workers in Greece found that the prevalence of quiet quitting among nurses was 67.4%, higher than among physicians (53.8%) and other professional groups (40.3%), indicating that nursing represented the largest share of this behavior across the groups assessed [10]. In another study conducted in the same country, focusing exclusively on nurses, the overall quiet quitting score averaged above the proposed scale cut-off, suggesting that participants could be characterized as quiet quitters [11]. Although this behavior existed before the pandemic, COVID-19 appears to have intensified it by exposing structural weaknesses and worsening working conditions, thereby increasing the frequency of professional disengagement [12].

More than just emergent behavior, quiet quitting can be interpreted as a manifestation of defensive adaptation in response to demanding and prolonged exhausting working conditions [6]. This association can be interpreted within the Job Demands–Resources (JD-R) model, which posits that excessive work demands, when combined with insufficient resources, tend to deplete workers’ energy and weaken behaviors associated with professional engagement [13,14]. In the context of nursing, chronic work overload, staff shortages, low recognition, and limited management support can deplete nurses’ physical, emotional, and cognitive resources, increase burnout, and promote quiet quitting as a limited and self-protective form of disengagement [14,15,16]. Besides its individual effects, burnout among nursing professionals is recognized as an organizational and systemic issue, linked to absenteeism, team overload, reduced productivity, and a growing shortage of qualified professionals, thereby threatening the sustainability of health services [17].

Understanding this articulation also requires recognizing burnout as a multidimensional phenomenon that includes physical fatigue, cognitive fatigue, and emotional exhaustion, reflecting the gradual depletion of essential resources needed to maintain performance, attention, and personal investment in work over time. In this context, physical and cognitive fatigue can impair nurses’ proactivity, care coordination, and relational availability, negatively impact professional performance, and increase the likelihood of care-related errors. At the same time, emotional exhaustion may lead to distancing, apathy, and reduced involvement in workplace interactions, aligning with the lack of motivation and disinterest observed in quiet quitting [18,19]. Therefore, quiet quitting may be a practical expression of this exhaustion, where professionals limit their effort to what is strictly necessary, removing extra initiatives and contributions as a self-protection measure in response to ongoing demands and limited recovery [11].

Together, quiet quitting and burnout pose significant challenges for nursing leadership. In high-pressure environments with workforce shortages, leaders must protect care quality and patient safety, ensure continuity of care, and promote workforce sustainability. Resource constraints and poor working conditions are associated with higher workloads, dissatisfaction, and negative outcomes, thereby affecting workforce stability and care quality [20]. Furthermore, when these issues occur, it becomes harder to organize collective efforts, improve interprofessional teamwork, and maintain effective care coordination—elements that rely heavily on an organized workplace, clear communication, and psychologically safe environments [6,21].

Despite the growing interest in the relationship between quiet quitting and burnout, the literature remains limited regarding the integrated analysis of these phenomena, the conceptual distinction between quiet quitting and related constructs, and the understanding of how specific dimensions of burnout are associated with specific domains of quiet quitting in the nursing context, considering both sociodemographic and occupational characteristics. In this sense, the present study aimed to analyze the relationship between quiet quitting and burnout among nurses, while considering the influence of sociodemographic and occupational factors in healthcare settings.

## 2. Materials and Methods

### 2.1. Study Design

This was a quantitative, observational, analytical cross-sectional study, reported in accordance with the Strengthening the Reporting of Observational Studies in Epidemiology (STROBE) Statement [22].

### 2.2. Setting and Participants

Data collection occurred between April and July 2025 at a local health facility in northern Portugal. Local health units are public institutions that provide integrated primary and hospital care, coordinated across different levels of service and focused on citizen-centered care.

This local health unit comprises two groups of health centers, focused on primary healthcare, and two hospitals. One hospital is centrally located and serves as a national reference for healthcare, training health professionals, research, and innovation. The other hospital functions as a backup facility, offering outpatient and rehabilitation services.

All nurses working in primary healthcare and hospital departments, including medicine, surgery, women’s and children’s health, and emergency services, were deemed eligible to participate. A non-probability convenience sampling method was employed, aiming to include the pool of nurses available at the participating facilities during data collection. Nurses with less than six months of professional experience and those absent from work for an extended period due to leave or medical reasons were excluded.

### 2.3. Data Collection Instruments

A two-part questionnaire served as the data collection tool. The first part included questions designed to profile participants’ sociodemographic, professional, and academic backgrounds. These questions covered gender, marital status, age, total professional experience, current work experience, type of employment contract, professional category, and work schedule.

In the second part of the questionnaire, the Portuguese versions of the Quiet Quitting Scale (QQS-PT) [23] and the Shirom–Melamed Burnout Measure (SMBM) [24] were administered.

#### 2.3.1. Quiet Quitting Scale (QQS-PT)

The QQS was originally developed by Galanis et al. (2023) [5], and adapted and cross-culturally validated for the Portuguese population by Ventura-Silva et al. (2025) [23], showing strong psychometric properties. In the Portuguese validation study, Cronbach’s global alpha was 0.918, and McDonald’s omega was 0.922, indicating excellent internal consistency. This instrument assesses silent abandonment, understood as the psychological distancing and intentional limitation of workers’ involvement in their contractual obligations within the organization. The content of the example items covers emotional distance from work, reduced initiative to go beyond assigned tasks, and decreased motivation to invest additional effort [5,23].

The scale comprises nine items distributed across three domains: Detachment/Disinterest (4 items), Lack of Initiative (3 items), and Lack of Motivation (2 items). A five-point Likert scale (1 = strongly disagree or never; 5 = strongly agree or always) is used as the response system. The total QQS score is obtained by summing all item scores and dividing the result by the total number of valid responses, with higher scores indicating a greater tendency towards silent abandonment [5,23].

#### 2.3.2. Shirom–Melamed Burnout Measure (SMBM)

The Shirom–Melamed Burnout Measure (SMBM) was developed by Shirom and Melamed (2006) [24] and, in the Portuguese context, adapted and validated by Gomes (2012) [25]. This instrument is structured around three dimensions: physical fatigue, which indicates feelings of work-related physical tiredness and reduced physical energy; cognitive fatigue, which manifests as mental exhaustion and a reduced ability to think and concentrate; and emotional exhaustion, characterized by feelings of emotional drain in relationships with others and a decrease in warmth and sensitivity towards their needs. An example of an item refers to feeling physically, mentally, or emotionally exhausted in relation to work [24,25].

It comprises 14 items, distributed across the three dimensions mentioned above. Responses are rated on a seven-point Likert scale, with one indicating Never and seven indicating Always. The score is calculated by summing the items of each dimension and then dividing the total by the number of items related to that subscale. It is also important to note that scoring can be done overall or by dimension, enabling the assessment of participants’ fatigue levels. Higher scores reflect greater physical fatigue, emotional exhaustion, and cognitive fatigue [25]. However, because these values reflect only burnout-related feelings, scores of 5 or higher on the Likert scale (Sometimes) may indicate problems in this domain [24].

### 2.4. Data Collection Procedures

After scheduling based on professionals’ availability in advance, the researchers visited various care settings and, with the nurse managers, provided each participant with an informed consent form and the data collection instrument. Participants received two unmarked envelopes to separately place the signed consent form and the completed questionnaire. After explaining the study’s objectives and procedures, participants signed the consent form, indicating their agreement to participate. Confidentiality and anonymity were maintained in the use and distribution of the collected data.

### 2.5. Data Analysis

For data analysis, descriptive and inferential statistics were used according to the nature of the variables and the objectives of the study, using the R software, version 4.4.1 (R Foundation for Statistical Computing, Vienna, Austria), in the RStudio environment, version 2024.04.2+764. Categorical variables were described by absolute and relative frequencies, and continuous variables by mean, standard deviation, minimum, and maximum values. The distribution of the main continuous variables (quiet quitting and burnout) was assessed using the Kolmogorov–Smirnov test. In the absence of normality, the associations between quiet quitting and burnout (global and domain scores) were examined using Spearman’s correlation coefficient (ρ) and corresponding *p*-values.

For multivariate analyses, multiple linear regression models were used to examine the relationship between burnout and quiet quitting, controlling for potential confounding factors. Covariate selection was guided by literature and its potential confounding role, including sex, age, marital status, education, length of service, and work context. Quiet quitting was defined as the mean scale score (range 1–5), encompassing an overall score and three domains: detachment/disinterest, lack of initiative, and lack of motivation. Burnout was defined as the mean SMBM score (range 1–7), covering an overall score and three domains: physical fatigue, cognitive fatigue, and emotional exhaustion. The reverse-worded items on the quiet quitting scale were recorded before score calculation so that higher scores consistently indicated higher levels of the construct. Four regression specifications were tested: (A) total burnout as a predictor of overall quiet quitting; (B) burnout domains, introduced simultaneously, as predictors of overall quiet quitting; (C) total burnout as a predictor of each quiet quitting domain in separate models; (D) Burnout domains as predictors of each quiet quitting domain, also in separate models. The analyses followed a complete case study approach. Length of service was not retained in the final models due to collinearity with age, identified by variance inflation factors.

Before modeling, multicollinearity, heteroscedasticity, and the presence of influential observations were assessed. Since heteroscedasticity was detected in the preliminary diagnoses (Breusch–Pagan test), the regression coefficients were presented with robust standard errors consistent with heteroscedasticity (HC1). Multicollinearity was assessed using variance inflation factors, and model fit was summarized using the coefficient of determination (R^2^). The influence diagnoses identified one observation with high leverage; therefore, sensitivity analyses excluding this observation were performed to assess the robustness of the results. The level of statistical significance was set at *p* < 0.05.

### 2.6. Ethical Approval and Informed Consent

The Health Research Ethics Committee approved the study under opinion number 51-2025. Subsequently, the hospital’s Board of Directors approved the data collection and granted authorization.

Nurses who agreed to participate signed the informed consent form, and confidentiality and anonymity were preserved. Participants placed their complete questionnaires in unmarked envelopes, which were then collected by the researchers.

The present study is part of a wider research project entitled “Quiet Quitting na Enfermagem: Como o Burnout e os Conflitos Trabalho-Família Influenciam a Intenção de Sair”.

## 3. Results

A total of 1097 nurses participated in the study, with a predominance of female participants (84.0%). The mean age was 42.1 years (SD = 9.6), ranging from 23 to 66 years, and regarding marital status, most participants reported being married or in a non-marital partnership (64.5%). In terms of education, most held a bachelor’s/undergraduate degree (84.6%), while 15.0% had a master’s degree (Table 1).

Regarding the occupational profile, most participants worked in a hospital setting, particularly in medical/clinical services (33.5%) and surgical services (29.1%). Concerning professional role, staff/clinical nurses accounted for the majority (63.4%), followed by specialist nurses (33.7%), with only 2.8% working as nurse managers. The mean time for professional experience was 18.9 years (SD = 9.6), and the mean time working in the current service was 11.6 years (SD = 8.8). Notably, among specialist nurses (n = 368), the mean duration of practice as a specialist was 8.8 years (SD = 6.0), and among nurse managers (n = 31), the mean was 14.4 years (SD = 7.1) (Table 1).

Participants presented a mean overall quiet quitting score of 2.5 (SD = 0.5), ranging from 1.8 to 4.1. Across dimensions, mean scores were 2.2 (SD = 0.6) for detachment/disinterest, 2.7 (SD = 0.4) for lack of initiative, and 3.0 (SD = 1.1) for lack of motivation. Regarding burnout (SMBM), the overall mean score was 3.8 (SD = 1.3), ranging from 1 to 7, with the highest mean observed for physical fatigue (4.8; SD = 1.5), followed by emotional exhaustion (3.9; SD = 1.6) and cognitive fatigue (2.8; SD = 1.3) (Table 2).

Spearman’s correlation analyses revealed positive associations between overall quiet quitting and total burnout (ρ = 0.463; *p* < 0.001), as well as with physical fatigue (ρ = 0.423; *p* < 0.001), cognitive fatigue (ρ = 0.408; *p* < 0.001), and emotional exhaustion (ρ = 0.450; *p* < 0.001). The detachment/disinterest domain also showed positive correlations with all burnout indicators, with coefficients ranging from 0.139 to 0.266 (all *p* < 0.001). Lack of motivation revealed the strongest positive associations, with coefficients ranging from 0.475 to 0.588 (all *p* < 0.001). In contrast, lack of initiative showed weak negative correlations with total burnout, physical fatigue, and cognitive fatigue, with no statistically significant association observed with emotional exhaustion (Table 3).

In Model A, total burnout showed a positive association with overall quiet quitting, even after adjusting for the covariates included in the model (β = 0.155; robust standard error = 0.009; t = 16.378; *p* < 0.001; R^2^ = 0.250). In Model B, when the burnout domains were introduced simultaneously, physical fatigue (β = 0.131; robust standard error = 0.020; t = 6.536; *p* < 0.001) and cognitive fatigue (β = 0.142; robust standard error = 0.024; t = 5.880; *p* < 0.001) remained significantly associated with overall quiet quitting, while emotional exhaustion did not show a statistically significant association (β = 0.034; robust standard error = 0.025; t = 1.342). This model explained 34.1% of the variability in overall quiet quitting (Table 4).

In Model C, total burnout was positively and statistically significantly associated with the three dimensions of quiet quitting: detachment/disinterest (β = 0.277; *p* < 0.001), lack of initiative (β = 0.199; *p* < 0.001), and lack of motivation (β = 0.472; *p* < 0.001). The respective models explained 22.0%, 18.8%, and 37.0% of the observed variability in these domains (R^2^ = 0.220; R^2^ = 0.188; R^2^ = 0.370, respectively). In Model D, detachment/disinterest showed statistically significant associations with both physical and cognitive fatigue; lack of initiative was also associated with both physical and cognitive fatigue; and lack of motivation was associated with physical fatigue and emotional exhaustion. The association between cognitive fatigue and lack of motivation was at the threshold of statistical significance (*p* = 0.053). In these domain-specific models, the R^2^ values ranged from 0.189 to 0.387. Sensitivity analyses, conducted after excluding the observation with high leverage, produced virtually identical estimates for the overall quiet quitting model.

Taken together, the regression results indicated that the overall quiet quitting score was associated with burnout, and that the pattern of these associations varied across the construct’s dimensions, with lack of motivation showing the highest standardized coefficient.

## 4. Discussion

Quiet quitting is especially relevant in nursing because this profession requires ongoing effort not only technically, but also relationally and discretionary. Looking at these results in light of the Job Demands and Resources (JDR) model helps to understand why this behavior can emerge in highly demanding care contexts: when workload and organizational pressures are high, and support resources prove insufficient, energy depletion tends to intensify, making it more difficult to maintain behaviors associated with professional engagement [13,14,16]. From this perspective, quiet quitting should not be equated with burnout or cynicism but rather understood as a behavioral manifestation of limited investment in work. Unlike cynicism, which reflects a more negative psychological stance towards professional activity, quiet quitting is characterized mainly by the restriction of discretionary effort while still maintaining compliance with the formal requirements of the role [7,8,14].

Since most participants hold a bachelor’s degree, this result might reflect a reality characterized by limited opportunities for academic advancement and professional growth. The literature shows that a lack of developmental opportunities and academic or professional recognition is linked to decreased emotional engagement at work and increased emotionally detached behaviors [7]. The fact that few nurses hold management positions may also indicate a potential leadership gap, since positive leadership styles are described in the literature as organizational resources linked to lower burnout and greater work engagement [13]. This issue may be especially relevant in high-intensity hospital care services, such as Medicine and Surgery, where emotional and work pressures are intense. Studies also indicate that high workloads are associated not only with burnout but also with increased quiet quitting [16].

A Chinese study involving 778 hospital nurses found that support, resources, opportunities, and information, collectively called structural empowerment, along with feelings of optimism, hope, efficacy, and resilience, predict work engagement. This engagement appeared as increased dedication, vigor, and absorption among the nursing staff [26].

The highest burnout dimension score is Physical Fatigue (M = 4.8), followed by Emotional Exhaustion (M = 3.9). This value of 4.8 is particularly concerning as it exceeds the midpoint of the scale, although these results align with international post-pandemic studies, which demonstrate that the physical workload and the scarcity of human resources in hospital units (where the majority of our sample is concentrated) result in profound somatic wear and tear [27,28,29]. In Portugal, studies have already warned that Portuguese nurses show higher levels of physical exhaustion than the European average due to long working hours [30,31,32].

Quiet quitting had an overall average score of 2.5; however, the “Lack of Motivation” dimension (M = 3.0) stands out as the main component of this phenomenon in the sample. Research indicates that in a toxic work environment or one lacking recognition, nursing professionals may choose to perform only what is strictly contracted [33,34,35]. A score of 3.0 in “Lack of Motivation” may suggest some erosion of vocational connection and discretionary proactivity, though this interpretation should remain cautious and context-dependent.

It is important to highlight that physical and cognitive fatigue displayed the most consistent independent associations with silent abandonment of work, which aligns with nursing contexts where sustained workload, interruptions, surveillance, and decisional density can limit the energy available for initiative, coordination, and extra-functional effort [17,36,37,38,39]. In turn, emotional exhaustion revealed weaker or non-independent associations in the adjusted models. One possible explanation is that its variance was shared with physical and cognitive fatigue, which was considered when the three domains were introduced simultaneously; another is that emotional exhaustion may be more context-dependent and more related to interpersonal tension than to the behavioral limitation of discretionary effort measured by the silent abandonment scale. Although these associations should not be interpreted causally, they can help identify areas of burnout that deserve attention in nursing management, namely fatigue management, staffing adequacy, recovery opportunities, and supportive supervision.

Although closely related, burnout and silent abandonment are not equivalent phenomena. Burnout is configured as a psychosocial syndrome associated with occupational exhaustion, while silent abandonment represents a behavioral response characterized by the intentional limitation of involvement beyond what is strictly necessary [8]. In the present study, the associations observed are consistent with the interpretation that burnout and silent abandonment coexist as related dimensions of stress and limited involvement, particularly when physical and cognitive energy for professional investment becomes limited.

It is also important to note that the explanatory models showed R^2^ values ranging from 22.0% to 38.7%, indicating that burnout-related indicators accounted for a substantial proportion of the variability in quiet quitting, especially in the “Lack of Motivation” dimension. However, a significant portion of the variability remains unexplained, suggesting that other organizational, relational, and individual factors, such as organizational climate and justice, social support, and professional satisfaction, may also play a role in this phenomenon, underscoring its multifactorial nature [16,40].

The results for the ‘Lack of Initiative’ dimension warrant careful consideration, as this domain showed weak and slightly negative bivariate correlations with burnout syndrome, but positive associations in the adjusted models. This pattern should not be interpreted as evidence of an intrinsically unreliable subscale, as the Portuguese validation study reported acceptable internal consistency for this domain (Cronbach’s α = 0.788, approximately 0.80; McDonald’s ω = 0.852) [23]. Instead, the contradictory pattern observed here may reflect contextual characteristics of nursing work, statistical suppression after covariate adjustment, or the possibility that a minimal level of operational initiative is maintained despite high levels of exhaustion. Taken together, these findings suggest that quiet quitting may manifest itself more clearly through emotional and motivational detachment than through a simple linear reduction in visible initiative.

The findings of this study also support previous research that emphasizes the importance of nursing leadership in preventing quiet quitting and workplace suffering. Leadership plays a key role in mitigating quiet quitting by fostering communication and strengthening a sense of community at work, even in hostile work environments. Dialogical communication and enhanced organizational interactions increase engagement and help decrease this phenomenon [41].

Quiet quitting should not be seen solely as a dysfunction. In certain contexts, it can also represent an adaptive strategy for setting boundaries or a form of everyday resistance to chronic overload, lack of recognition, or unsustainable expectations. This interpretation is particularly relevant in nursing, where professionals may continue to provide essential care, only withdrawing from activities that exceed the scope of their duties to protect their limited personal resources [7,8,33]. Furthermore, the meaning and visibility of quiet quitting are likely influenced by culture and professional norms. Depending on the type of healthcare system, this behavioral pattern may manifest more subtly, while in other contexts it may be more explicitly framed as setting boundaries or refusing unpaid extra-functional tasks [42]. This cultural contingency should moderate how broadly we generalize our conclusions beyond this specific Portuguese public sector context, as the meaning and expression of quiet quitting can differ across organizational setups and cultural expectations.

The reduction in professional engagement compromises both the development of the nurse and the performance of healthcare organizations by limiting the incorporation of innovative practices and the continuous improvement of care [43], which can be understood as an early indicator of organizational weaknesses, signaling work environments that are not conducive to engagement, innovation, and professional retention [40].

The association between physical and cognitive fatigue and reduced professional motivation is particularly relevant in nursing, as these factors may affect clinical vigilance, decision-making, and the quality of care [38,39]. Thus, quiet quitting, when it occurs alongside burnout, may represent not only an organizational concern but also a potential patient-safety issue, especially in high-complexity hospital settings.

In practice, the current findings should be interpreted cautiously and not seen as direct evidence of the effectiveness of interventions. Still, the literature suggests that regular, in-depth conversations with professionals to understand expectations, needs, and career aspirations, along with greater alignment between organizational and individual goals, development opportunities, and the prevention of abusive or authoritarian leadership, may constitute relevant strategies to address disengagement over time [44].

In this sense, more engaging leadership is negatively associated with quiet quitting and positively associated with work engagement. Authors state that nursing managers should adopt leadership styles that promote motivation and participation to improve clinical outcomes and reduce disengagement, providing direct evidence that managerial behavior is a protective factor against quiet quitting [40,45].

Therefore, shared leadership and professional development can serve as protective organizational resources, particularly in contexts where leadership climate, retention, and work-related distress are closely intertwined. However, these implications should be interpreted with caution, since the present results stem from associations observed in a single organizational context. Institutional management and nurse leaders can benefit from actively listening to team experiences to identify context-sensitive actions that promote well-being and strengthen connection to the work environment. Besides the strategies discussed in the literature [26,44], locally tailored approaches that consider organizational culture could be especially important.

Since the “Lack of Motivation” dimension showed the strongest association with burnout, monitoring indicators of physical and cognitive wear and tear can help services identify possible early signs of professional disengagement. However, this possibility should be understood as hypothesis-generating, not prescriptive, requiring confirmation in longitudinal and intervention studies.

Finally, it is important to highlight some limitations of this study. First, its cross-sectional design prevents establishing causality or temporal direction between burnout and quiet quitting, so the results should be interpreted strictly in associative terms. Furthermore, the measures were self-reported, which may introduce social desirability and common method bias, potentially influencing the magnitude of the observed associations. Additionally, the “Lack of Initiative” dimension showed low internal consistency, requiring caution in interpreting results specific to this subscale. Finally, because the study was conducted in a single local health unit in northern Portugal, within a specific public and organizational context, the results may not be easily generalizable to other health organizations, regions, or health systems with distinct endowment models, leadership structures, professional norms, and cultural expectations. This limitation is particularly relevant because quiet quitting is likely to be sensitive to organizational climate and cultural context.

## 5. Conclusions

More than just an isolated phenomenon, quiet quitting revealed a moderate and consistent association with burnout among nurses in Portugal’s healthcare context, even after adjusting for sociodemographic and occupational factors. The observed pattern was stronger for physical and cognitive fatigue and for the lack of motivation dimension of quiet quitting. These results suggest that quiet quitting may indicate stress and limited engagement in demanding nursing contexts, rather than simply reflecting a voluntary form of disengagement. Given the cross-sectional nature of the study, these interpretations should remain associated and cautious.

From an applied perspective, the results can help inform leadership and organizational reflection on workload management, recovery opportunities, recognition, and positive work environments, but should not be interpreted as directly transposable to specific interventions. Longitudinal and multicenter studies are essential to clarify temporal relationships, evaluate cultural differences, and determine whether changes in workplace resources correlate with burnout-related disengagement.

## Figures and Tables

**Table 1:** Sociodemographic, academic, and professional characteristics of the nursing sample (*n* = 1097).

Variable	
Gender	n (%)
Male	175 (16.0)
Female	922 (84.0)
Age	years
Mean (±SD)	42.1 (±9.6)
Minimum; maximum	23; 66
Marital status	n (%)
Not married	315 (28.7)
Married/nonmarital partnership	708 (64.5)
Divorced	69 (6.3)
Widower	5 (0.5)
Educational qualification	n (%)
Bachelor’s degree	4 (0.4)
Graduation	928 (84.6)
Master’s degree	165 (15.0)
Work context	n (%)
Primary health care, family health unit	82 (13.4)
Hospital healthcare, department of medicine service	367 (33.5)
Hospital healthcare, department of surgery service	319 (29.1)
Hospital healthcare, department of intensive care medicine	127 (11.6)
Hospital healthcare, department of women and children	137 (12.5)
Condition of exercise of the profession	n (%)
Nurse	696 (63.4)
Specialist nurse	370 (33.7)
Manager nurse	31 (2.8)
Specialty area (n = 392)	n (%)
Medical-surgical nursing	96 (8.9)
Rehabilitation nursing	119 (10.8)
Mental Health and Psychiatric Nursing	16 (1.5)
Maternal and obstetric health nursing	37 (3.4)
Pediatric and child health nursing	59 (5.4)
Community nursing	65 (6.0)
Time of professional experience in the profession	years
Mean (±SD)	18.9 (±9.6)
Minimum; maximum	1; 43
Time of professional practice in the current service	years
Mean (±SD)	11.6 (±8.8)
Minimum; maximum	1; 43
Time of professional practice as a specialist nurse (n = 368)	years
Mean (±SD)	8.8 (±6)
Minimum; maximum	1; 39
Time of professional practice as a manager nurse (n = 31)	years
Mean (±SD)	14.4 (±7.1)
Minimum; maximum	1; 24

Note: SD = standard deviation; n = absolute frequency; % = relative frequency.

**Table 2:** Descriptive statistics of the instruments and their dimensions (mean, standard deviation, and minimum–maximum values) (*n* = 1097).

Variable	Mean (SD)	Minimum; Maximum	Cronbach’s
Quiet Quitting Scale—General	2.5 (0.5)	1.8; 4.1	0.8
Detachment/Disinterest	2.2 (0.6)	1; 4.2	0.8
Lack of Initiative	2.7 (0.4)	1; 4	0.8
Lack of Motivation	3.0 (1.1)	1; 5	0.9
Shirom–Melamed Burnout Measure—General	3.8 (1.3)	1; 7	0.9
Physical Fatigue	4.8 (1.5)	1; 7	0.9
Cognitive Fatigue	2.8 (1.3)	1; 7	0.9
Emotional Exhaustion	3.9 (1.6)	1; 7	0.9

Note: SD = Standard deviation.

**Table 3:** Spearman correlations between Quiet Quitting Scale (overall and dimensions) and the Shirom–Melamed Burnout Measure (overall and domains) (*n* = 1097).

Variable	Smbm-Total	Smbm-PF	Smbm-CF	Smbm-EE
Quiet Quitting Scale—General	0.463 (<0.001)	0.423 (<0.001)	0.408 (<0.001)	0.450 (<0.001)
Detachment/Disinterest	0.201 (<0.001)	0.139 (<0.001)	0.167 (<0.001)	0.266 (<0.001)
Lack of Initiative	−0.082 (0.007)	−0.124 (<0.001)	−0.075 (0.013)	−0.008 (0.779)
Lack of Motivation	0.583 (<0.001)	0.588 (<0.001)	0.532 (<0.001)	0.475 (<0.001)

Note: Smbm-total = Shirom–Melamed Burnout Measure—General; Smbm-PF = Shirom–Melamed Burnout Measure—Physical Fatigue; Smbm-CF = Shirom–Melamed Burnout Measure—Cognitive Fatigue; Smbm-EE = Shirom–Melamed Burnout Measure—Emotional Exhaustion.

**Table 4:** Robust regression models examining the association between burnout (SMBM) and quiet quitting (overall and dimensions) (*n* = 1097).

Model A					
Predictor		*β*	Robust SE	*t*	*p*
SMBM—Total		0.155	0.009	16.378	<0.001
Sex (category 2 vs. ref.)		−0.145	0.037	−3.941	<0.001
Age (years)		0.001	0.005	0.303	0.762
Time in profession (years)		−0.002	0.005	−0.380	0.704
Time in service (years)		0.001	0.002	0.805	0.421
Model fit: R^2^ = 0.250					
Model B					
Predictor (SMBM domains)		*β*	Robust SE	*t*	*p*
Physical fatigue		0.131	0.020	6.536	<0.001
Cognitive fatigue		0.142	0.024	5.880	<0.001
Emotional exhaustion		0.034	0.025	1.342	0.180
Adjusted for: sex, age, marital status, education, time in profession, time in service, work context.					
Model fit: R^2^ = 0.341					
Note: Sensitivity analysis excluding one high-leverage observation (case 720; N = 1096) yielded virtually identical estimates (PF β = 0.131; CF β = 0.142; EE β = 0.034).					
Model C					
Predictor (SMBM—Total)		*β*	Robust SE	*t*	*p*
Detachment/Disinterest		0.277	0.019	14.27	<0.001
R^2^ = 0.220					
Lack of Initiative		0.199	0.014	14.71	<0.001
R^2^ = 0.188					
Lack of Motivation		0.472	0.021	23.01	<0.001
R^2^ = 0.370					
Model D					
Outcome	Predictor (SMBM domain)	*β*	Robust SE	*t*	*p*
Detachment/Disinterest	Physical fatigue	0.071	0.026	2.73	0.007
	Cognitive fatigue	0.235	0.032	7.39	<0.001
	Emotional exhaustion	0.004	0.033	0.11	0.915
R^2^ = 0.234					
Lack of Initiative	Physical fatigue	0.092	0.022	4.10	<0.001
	Cognitive fatigue	0.068	0.026	2.58	0.010
	Emotional exhaustion	0.041	0.027	1.53	0.126
R^2^ = 0.189					
Lack of Motivation	Physical fatigue	0.310	0.031	9.85	<0.001
	Cognitive fatigue	0.071	0.036	1.94	0.053
	Emotional exhaustion	0.084	0.038	2.21	0.027
R^2^ = 0.387					

Note: β = Standardized regression coefficient; SE = Robust standard error; t = Test statistic; *p* = Statistical significance value (*p* < 0.001).

## Data Availability

The data presented in this study is available on request from the corresponding author. The data are not publicly available due to ethical restrictions.

## Public Involvement Statement

No public involvement in any aspect of this research.

## Guidelines and Standards Statement

This manuscript was prepared based on STROBE for observational study research.
